# Promoting Family/Friend Involvement in Care Planning in Care Homes: A Qualitative Exploration of the Usefulness and Relevance of an Information Resource

**DOI:** 10.1111/hex.70715

**Published:** 2026-06-04

**Authors:** Thaïs Caprioli, Jonathan Taylor, Yuri Hamashima, Alison Charles, Jacqueline Damant, Clarissa Giebel, Michele Peters, Madalina Toma, Anna Ferguson Montague, Lynne Wright, Nick Smith

**Affiliations:** ^1^ Department of Primary Care and Mental Health University of Liverpool Liverpool UK; ^2^ NIHR ARC North West Coast University of Liverpool Liverpool UK; ^3^ Nuffield Department of Population Health University of Oxford Oxford UK; ^4^ The National Institute for Health and Care Research Applied Research Collaboration West (NIHR ARC West) at University Hospitals Bristol and Weston NHS Foundation Trust Bristol UK; ^5^ Population Health Sciences, Bristol Medical School University of Bristol Bristol UK; ^6^ Centre for Health Services Studies University of Kent Canterbury UK; ^7^ Care and Policy Evaluation Centre, London School of Economics London UK; ^8^ Patient, Carer and Public Involvement and Engagement Advisor UK

**Keywords:** care homes, care planning, health information, information resources, older adults

## Abstract

**Background:**

The involvement of family/friends in the care planning of an older adult residing in a care home can help to foster person‐centred care. This study explored and incorporated family/friends' views on the relevance and usefulness of a draft information resource focusing on care planning to address and support older adults' needs.

**Methods:**

The research team drafted an information resource to help family/friends better understand care planning. The contents of the draft resource were informed by previous research on care planning in older adult care homes, which included a scoping review, a modified Delphi survey and consultations with health and social care professionals. Feedback on the draft resource was collected through remote focus group discussions with family/friends of older adults residing in care homes in England in November 2024. Participants were invited to share their views on the draft information resource and if/how it could be improved. Audio recordings were transcribed verbatim and the data thematically analysed in NVivo.

**Findings:**

The views of 28 family/friends were obtained across four focus group discussions. Three main themes were generated: experiences with care planning, usefulness of the information resource and improving and sharing the information resource. Findings depict a heterogeneity in awareness of, and involvement in, care planning. Most participants perceived the information resource to have the potential to raise awareness of care planning and promote family/friend involvement. Subject to changes, several participants said that they would share the document. Some, largely due its presentation, said they would not.

**Conclusions:**

Most participants believed that an information resource on care planning would be helpful. Future research is required to explore whether the document leads to increased family/friend involvement in care planning.

**Patient or Public Contribution:**

Two family members with experience of supporting a relative in a care home were involved in devising and revising the different iterations of the information resource and topic guide, and in interpreting findings.

## 1 Background

Over 250,000 people aged over 65 live in care homes, with or without nursing care, in England [1]. Trends suggest that, within the last two decades, older adults residing in care homes are experiencing an increasing number of complex care and support needs [2]. While the type and degree of support required varies over time and between individuals, domains of needs may include promoting independence and autonomy, assisting with completing daily living activities and supporting social and emotional well‐being [3, 4].

The development of a care plan, that is, a document that outlines a person's needs and how these will be met [5], should guide the delivery of their care and support. The Care Act 2014 (https://www.legislation.gov.uk/ukpga/2014/23/contents) stipulates that local authorities in England must conduct assessments where individuals may have a need for care and support. Moreover, the Care Quality Commission (CQC), the public body that regulates health and social care providers in England, states that care providers, including care homes, are required to work in partnership with individuals they support to ensure their involvement ‘…in the planning, management and review of their care…’ [6]. The involvement of individuals and/or those lawfully acting on their behalf is designed to promote person‐centred care by facilitating the delivery of care and support that meets individuals' needs while, where possible, reflecting their personal preferences [6]. Care planning should be an iterative process whereby agreed outcomes for the delivery of care and support are reviewed and the views of individuals' and/or those lawfully acting on their behalf are actively sought and acted upon [6, 7, 8]. The wider care planning process can include addressing current and continuing care and support needs, and planning for future care [9]. The latter is known as advance care planning (ACP) and focuses on identifying a person's preferences and priorities relating to future care [9]. Henceforth, we employ the term ‘care planning’ to refer care planning to address current care and support needs and use the term ‘ACP’ to discuss planning for future care.

Whether consented by the resident or legally appointed under the Mental Capacity Act (2005) (https://www.legislation.gov.uk/ukpga/2005/9/contents), the level of family/friend engagement in care planning is often influenced by pre‐existing relationships and prior contributions to a person's care [10]. The involvement of family/friends in care planning can help to facilitate the delivery of person‐centred care by ensuring that health and social care professionals are aware of a person's preferences, interests and routines [11, 12, 13]. Family/friend involvement is valued by older adults [14], advocated by health and social care professionals [12] and is considered a ‘key principle’ to care planning [15, 16]. However, the level of involvement of family/friends in care planning varies across care homes [12], and challenges have been documented. These include facilitating conversations with family/friends who have limited involvement in the residents' life and/or who hold different views relating to care priorities [10, 13], insufficient staff training and the need to prioritise direct care due to finite resources [12]. The benefits and barriers to involvement experienced by family/friends have largely been explored in relation to ACP. While involvement in ACP is associated with a reduced decision‐making uncertainty and a more positive opinion of their relative's care [17, 18, 19, 20, 21], concerns about the future and limited awareness of the process were found to hinder family/friend engagement [22].

Providing information resources to family/friends has been identified as an approach to help raise awareness and promote involvement in ACP [22, 23]. These resources—including videos, websites, and print materials like pamphlets, booklets and brochures [24, 25]—often encourage the exploration of goals and values, support decision‐making, facilitate reflection on care and treatment preferences and/or aid in the communication or documentation of these preferences [25, 26]. Information resources on ACP, in the form of pamphlets and booklets, have been perceived as useful by family/friends [27, 28], encouraged reflection on future care and increased comfort in discussing end‐of‐life care [28]. However, while the use such information resources facilitated reflection on future care, due to an inclination to protect one another from the emotional topic, end‐of‐life preferences were seldom discussed between family/friends and the person residing in a care home [28]. Little is known about whether information resources on care planning, as opposed to solely on ACP, would help to promote improved family/friend involvement.

Although some websites, including charities (e.g., [29, 30, 31]), health and social care [5, 8] and private social care organisations (e.g., [32]), provide information on care planning in England, not all appear to be specific to older adults residing in care homes. Therefore, informed by previous research, we drafted an information resource on care planning, written for family/friends and sought to explore and incorporate their views on the relevance and usefulness of the document.

## 2 Methods

An explorative descriptive qualitative design was employed. The views of family/friends on the relevance and usefulness of the information resource were explored through focus group discussions. Focus groups were chosen to enable the exploration of a breadth of views on a specific topic, and the interaction between participants can provide useful information about the degree of consensus and variety among them [33]. This study forms part of a larger project, ‘Wellbeing in Care Homes’ (https://arc-kss.nihr.ac.uk/npp-adult-social-care-social-work/our-programme-of-work/well-being-in-care-homes-implementation-of-outcomes-based-care-planning), that seeks to improve the wellbeing of older adults residing in care homes in England by promoting the adoption of care planning approaches which focus on quality of life. Reporting followed the Standards for Reporting Qualitative Research [34] (Supporting Information: File S1).

### 2.1 Development of the Draft Information Resource

The draft document developed for family/friends was informed by earlier phases of the ‘Wellbeing in Care Homes’ project, which included: a scoping review of care planning interventions [23]; consultations with health and social care professionals to understand how care planning is conducted in older adult care homes [12] and a modified Delphi survey to gain consensus on a set of ‘key principles’ to inform professionals' approach to care planning [15, 16]. Subsequently, the wider research team, including public advisors, identified the most pertinent ‘key principles’ to care planning related to family/friends. This information served as the basis for a draft information resource which drew together the key points in clear language and short sentences structured around a series of headings and bullet points (Supporting Information: File S2). The draft information resource was presented as a two‐sided Word document comprised of seven sections, which sought to introduce care planning and encourage family/friend involvement. While the draft information resource focused on care planning to address older adults' current care and support needs, ACP forms an important aspect of wider care planning process, and therefore, was included.

### 2.2 Participants and Recruitment

People supporting a family member or friend aged over 65 living in care home (nursing or residential) in England within the past 12 months were eligible to participate. Awareness of and/or previous involvement in the care planning of the person they supported or had supported did not form part of the inclusion criteria. To minimise recall bias, people with a family member/friend who had not lived in a care home within the last 12 months were ineligible.

We employed a convenience approach to sampling. The research opportunity was shared through a flyer and emails to organisations supporting family carers, to care home staff who had participated in the modified Delphi survey and intermediary organisations, such as the Enabling Research in Care Homes (ENRICH) (https://enrich.nihr.ac.uk/) network, as well as by presenting at a patient and public involvement and engagement (PPIE) group. The study was also advertised on Join Dementia Research (https://www.joindementiaresearch.nihr.ac.uk/). Interested family/friends emailed members of the research team (J.T., Y.H. or T.C.), who arranged a phone call to explain the study, answer any questions and confirm their eligibility.

Given the relatively narrow aims of this study and available resources, ahead of data collection, we estimated that four focus group discussions, comprising seven participants, would yield sufficient data to answer our research questions. Seven participants per focus group were thought to provide a breadth of views while being manageable [35].

### 2.3 Data Collection

Focus group discussions were held on Zoom in November 2024. The topic guide was devised with public advisors working on the project. It sought to elicit participants' views on the information resource and did not directly ask questions relating to their experiences of care planning (Supporting Information: File S3).

Written informed consent was obtained through Qualtrics (https://www.qualtrics.com/en-gb/) ahead of data collection. Participants' demographic information (sex, age range, ethnic group, relationship to the person living in care home) and previous involvement in care planning was collected prior to the focus group discussions, using a questionnaire on Qualtrics. To prepare for the focus group discussion, participants were asked to read the draft information resource and to consider aspects which were helpful, and, if relevant, how the document could be improved. Participants were allocated to a focus group depending on their availability.

Focus group discussions were facilitated by two or three researchers with experience in qualitative research (J.T., Y.H., A.C. & T.C.). Participants were asked to keep the contents of the discussion confidential, and ‘ground rules’ were explained at the beginning of each discussion. To aid ongoing reflexivity, field notes were recorded by one researcher and shared following each focus group. Details recorded included a summary of participants' views and any thoughts arising while observing the discussion. Researchers debriefed following each focus group and reflected on aspects that had gone well and, if relevant, those that could be improved.

All focus group discussions were audio recorded using the function within Zoom. The recordings were transcribed verbatim by a professional agency and were anonymised for analysis.

### 2.4 Participant Reimbursement

Participants were reimbursed with a £30 shopping voucher. The amount was calculated using the minimum wage per hour at the time of planning the study and reflected the time required to review the draft information resource and attend the focus group, approximately 2 h in total, plus £5 to cover internet costs.

### 2.5 Data Analysis

We undertook a thematic analysis to elicit descriptive accounts of participants' views on the information resource, on NVivo (version 14). Following the six phases of thematic analysis [36], members of the research team (J.T., Y.H., A.C. & T.C.) familiarised themselves with the data by reading the transcripts multiple times and generating initial codes deductively, drawing upon the research questions. Subsequently, an initial coding framework was developed collaboratively, applied to the transcripts and assessed by the members of the research team for credibility. The flexibility of a thematic analysis enabled members of the research team to move from a more deductive to an inductive approach, thus generating themes. The last phase comprised writing up the findings and selecting the most appropriate quotes to illustrate analytical points. Several steps were undertaken to strengthen the trustworthiness of our analysis. These included scheduling regular peer debriefing meetings to assist with research reflexivity and triangulating interpretations and returning to the raw data regularly throughout the analysis to ensure referential adequacy [37].

To revise the content of the draft information resource according to feedback received, several team‐wide meetings, including experienced care home researchers and public advisors (see PPIE section), were held to discuss each suggestion to determine if corresponding changes to the document would be made. Changes were made if they were feasible within the scope of the study and were likely to increase the knowledge acquired, likelihood of the information resource being read [38, 39] and whether the content was applicable across care settings. Owing to limited resources, changes were solely made to the content of the information resource. Feedback related to changes other than the content of the document, including presentation and dissemination methods, were recorded and will be addressed as part of future work. To systematically consider each suggestion and whether a change was needed and could be made, feedback was categorised as follows: ‘addressed’ (suggestion has been incorporated); ‘partially addressed’ (suggestion has been partially incorporated); and ‘not addressed’ (suggestion has not been incorporated).

### 2.6 PPIE

Two public advisors with experience of caring for a person residing in an older adult care home in England were actively involved throughout the study. They advised on the development of the draft and revised information resource, topic guide and interpretation of the findings.

### 2.7 Ethics

Ethical approval was obtained from the Division for the Study of Law, Society and Social Justice Research Committee Ethics Panel at the University of Kent (reference: 1006) on 9 April 2024, prior to commencing the study.

## 3 Findings

A total of 28 participants participated in the four focus group discussions, which lasted a mean of 58 min 20 s (range: 00:56:50–01:00:24). Our sample were largely female (*n* = 19; 67.9%), aged between 55 and 64 (*n* = 14; 50.0%) and from a White ethnic background (*n* = 23; 82.1%) At the time of data collection, 21 (75.0%) participants were supporting an older adult residing in a care home and most participants were supporting or had supported a parent (*n* = 18; 64.3%). Participants' family member/friend were living or had lived in a nursing (*n* = 13; 46.4%) or specialist dementia (*n* = 9; 32.1%) care home, with most located in the South East of England (*n* = 9; 32.1%), London (*n* = 6; 21.4%) and the North West of England (*n* = 5, 17.9%). Apart from one person, participants were aware of their family member/friend's care plan, although their level of involvement in the person's care planning differed (Table 1). Supporting Information: File S4 shows the relationship of participants to the person(s) they supported/were supporting.

**Table 1:** Summary of participants’ demographic information (*n* = 28).

	*n* (%)
Sex	
Female	19 (67.9)
Male	9 (32.1)
Age group	
25–34	4 (14.3)
35–44	2 (7.1)
45−54	3 (10.7)
55−64	14 (50.0)
65−74	4 (14.3)
75 or older	1 (3.6)
Ethnic group	
Black/African/Caribbean/Black British	1 (3.6)
Arab	1 (3.6)
White	23 (82.1)
Asian/Asian British	2 (7.1)
Mixed/multiple ethnic group	1 (3.6)
Supported/supporting their^a^	
Aunt/uncle	4 (14.3)
Grandparent	2 (7.1)
Parent	18 (64.3)
Friend	1 (3.6)
Sibling	1 (3.6)
Spouse	2 (7.1)
Other^b^	3 (10.7)
Type of care home^a^	
Nursing	13 (46.4)
Residential	8 (28.6)
Mix of nursing/residential	1 (3.6)
Specialist dementia	9 (32.1)
Location of care home^a^	
London	6 (21.4)
North East	2 (7.1)
North West	5 (17.9)
South East	9 (32.1)
South West	4 (14.3)
West Midlands	1 (3.6)
Prefer not to say	3 (10.7)
Missing	1 (3.6)
Level of involvement in care planning^a^	
Is/was unaware	1 (3.6)
Do/did not contribute	3 (10.7)
Rarely contribute/contributed	10 (35.7)
Occasionally contribute/contributed	6 (21.4)
Often contribute/contributed	11 (39.3)

^a^ Three family/friends were supporting/had supported two older adults. All other family/friends were supporting or had supported one older adult.

^b^ Friend's father, Aunt's husband, Husband's aunt.

**Figure 1:**
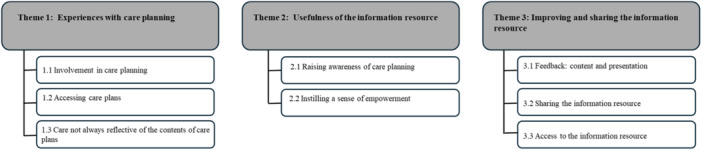
Theme and sub‐theme tree.

The following themes and sub‐themes were generated (Figure 1): experiences with care planning; usefulness of the information resource; and improving and sharing the information resource.

### 3.1 Experiences With Care Planning

#### 3.1.1 Involvement in Care Planning

While most participants were aware of care planning when participating in the focus groups, some were unfamiliar with care plans and/or that family/friends could ask to see the document.

My mum's been in a care home for four years now, and until this [research study] came up, I wasn't even aware what a care plan was.

FG3, P7

Well, just before this meeting [focus group], I thought that I would try to have a look at my dad's care plan, and I was surprised to learn that it's not that easy.

FG1, P5

Most participants recalled being asked questions or completing forms to help devise the initial care plan. However, at the time of being asked to contribute to the initial care plan, some participants demonstrated a limited understanding of care planning and its purpose.

We knew that there was a care plan, well we didn't know what it was, nobody told us what it was. We were asked to fill in a twenty‐page document with all details of our mum…

FG2, P4

Participants reported mixed experiences relating to their and their family member/friend's involvement in care planning. Some participants expressed satisfaction with their involvement and/or the person‐centred approach embodied.

…the care plan was a reflection of the care and attention which [name] was receiving, based on their knowledge of her, rather than being the framework into which [name] was placed…

FG2, P7

However, several participants reported limited involvement and were frustrated with their lack of meaningful participation, with some feeling excluded from important decisions made regarding the care of their family member/friend.

And it is true, they just take control of your loved ones and that's it, it's like, ‘butt out’, and it's absolutely heartbreaking when the system doesn't work.

FG4, P1

#### 3.1.2 Accessing Care Plans

Some participants were able to access care plans anytime, and when seen, were satisfied with the information recorded.

My sister is in a small specialist dementia unit…and her care plan is available in her room and it's there to see, and always has been.

FG3, P3

For many, the first step in trying to view the document involved speaking with care home staff. However, some participants perceived staff as reluctant to share the care plans and reported that they were often unable to access the care plan.

I was refused on numerous occasions access to my mum's care plan with various excuses.

FG1, P3

When care plans were accessed, some participants were not able to see the whole document or changes made following reviews were not clearly explained. Moreover, some participants reported on the inclusion of incorrect and/or omission of information.

And then found out that there were a lot of cut and pasted [in care plan], [name 1] was in it, whereas my mum's name is [name 2].

FG2, P4

Some participants did not feel the need to see the care plan. Reasons included being satisfied with the care received, not being the next of kin and/or a perception that the document was largely for care home staff. Nevertheless, participants reported that it would be valuable to have a condensed version of a person's care plan, which could be readily accessible to family/friends.

#### 3.1.3 Care Not Always Reflective of the Contents of Care Plans

Participants felt that care plans were not always a good indication of the care received. For example, sometimes the care received exceeded the contents of the care plans.

I have been to care homes [in a professional capacity] where the care plans weren't well written, but the care being provided was excellent.

FG3, P1

More often, however, participants felt that, while their family member/friend's care plan outlined their preferences, the information rarely informed the care provided. Examples included not adhering to a pressure sore treatment plan, failure to follow safety procedures and providing food that did not align with the person's preferences.

…I just think that the care plans are done essentially to be individualised, but then the care that people get is very generic.

FG4, P6

As such, some participants felt that their contributions to writing care plans were a ‘…waste of time’ (FG4, P1), and many viewed the process as a ‘tick‐box’ exercise largely to satisfy certain regulatory requirements.

… in reality it is about having things written down, to tick a box for CQC [Care Quality Commission], to tick a box for contractors and other people who are involved in procuring care….

FG1, P6

Some participants suggested factors that hindered the execution of care plans' contents. Issues within individual care homes, such the culture within the care home or inadequate communication amongst staff, were raised.

And I think that whether you devise the best care plan in the world, I'm not sure it'll make the blindest bit of difference unless you've got good communication between your team leaders or the management and the handing over between the staff.

FG4, P7

Issues experienced by individual care homes were thought to be compounded by broader challenges in the social care sector. These included high staff turnover, understaffing, dependence on agency staff, language barriers, inadequate pay and limited training.

…the turnover of staff, so they don't get familiar with the residents, and they don't know what's in the care plan … and maybe lack of training.

FG4, P6

### 3.2 Usefulness of the Information Resource

#### 3.2.1 Raising Awareness of Care Planning

Many participants believed that the information resource could address family/friends' unfamiliarity with care planning, notably during the earlier stages of supporting a person's transition into a care home.

I would say that this was a helpful document [information resource] … for anybody just going into this with a parent or brother or sister or whoever, not least because it will give them the idea that a care plan is something they ought to have. As [participant] has said, [they] didn't even know there should be one.

FG3, P4

A greater awareness of care planning and its purpose was seen by most participants as a prerequisite to encouraging family/friend involvement. The information resource was perceived as ‘…a bit of a guide as to what to expect…’ (FG2, P5) and how family/friends could become involved.

…I would definitely share it, and make people aware that they have the right to be able to see that document [care plan], and to change it if they've got lasting power of attorney, etc.

FG2, P6

Participants perceived that if family/friends understood the content of care plans, they could more effectively provide care home staff with pertinent information, especially when the initial plan was being devised.

It's quite good to see ‘oh that's the sort of thing they're [staff] looking for’ [to include in a care plan].

FG2, P4

One participant reported that, had they been more aware of the importance of a care plan, it would have enabled them to ‘reflect more on it and have the opportunity to input some more’ (FG2, P2).

### 3.3 Instilling a Sense of Empowerment

Some participants reflected on how the information resource would have empowered them to have been more engaged in care planning. For instance, participants commented on actions the document would have or had encouraged them to undertake, including asking how risks are being managed and asking to see a copy of the care plan.

I mean, after this conversation [focus group] we've had, I'll be going in tomorrow to ever so politely ask to see a copy of the care plan just in the office, even if they can't actually send it to me electronically.

FG1, P5

Following participation in this study, some participants reported that they planned to discuss their family member/friend's end‐of‐life preferences with care home staff, and to ensure these were known and taken into consideration.

…well years ago she had quite specific views about what she wanted, and sort of things like funeral arrangements, so I think next time I visit I'll speak to the staff and just, you know, make sure that kind of, her wishes are taken into consideration.

FG2, P5

Some participants also found that the information resource could be helpful for other family/friends to identify aspects of their family member/friend's care plan that need improving and to build their confidence to hold conversations with care home staff.

… as a relative knowing that I can use this document to kind of empower me to have those conversations. It would have been really helpful.

FG2, P2

Furthermore, participants commented that the information resource could help people make a more informed decision when identifying and selecting a care home that would best meet their family member/friend's care and support needs.

… this [information resource] should be like an onboarding questionnaire, ‘… I'll ask these following questions and see how they [care home staff] respond’. That is a document [information resource], you know, an aide memoire document to show the difference between where you're going to place your loved one.

FG3, P2

### 3.4 Improving and Sharing the Information Resource

#### 3.4.1 Feedback: Content and Presentation

Overall, participants expressed that the information resource was ‘… easy to understand…’ (FG4, P4) and comprised relevant and useful information. The glossary was reported to be ‘… very helpful as well’ (FG2, P5).

… overall it was a very good document, very well laid out, with different, you know, parts to it. Obviously, there are details within each section that one can incorporate, but very useful.

FG3, P1

Participants were encouraged to suggest changes to the draft information resource. Table 2 summarises participants' feedback regarding how the content of the information resource could be improved, and how it has been revised.

**Table 2:** Summary of participants' feedback regarding how the content the draft information resource could be improved and how it has been revised.

	Revising the draft information resource			
Feedback	Addressed	Partially addressed	Not addressed	Explanation of change/Revision
End‐of‐life preferences should be recorded in a document separate to the care plan.		x		Additional detail stating that end‐of‐life preferences may be recorded in a person's care plan, or depending on the care home, preferences may be recorded in a separate document, often known as an advanced care plan.
Include analysis of the legal aspects of care planning			x	This information resource intends to introduce the concept of care planning and how family/friends could be involved. It does not seek to provide legal advice or to analyse the legal aspects of care planning.
Discrepancy between the second paragraph and who is legally entitled to see the care plan in the ‘viewing a care plan’ section.	x			The discrepancy between sections has been amended.
Include information on the legal obligations of care homes around care planning		x		The information resource does not provide legal advice. Instead, it seeks to introduce care planning, and not all activities regulated by the CQC can be summarised. The link to the CQC website has been included within the ‘useful links’ section.
Outline who is accountable and responsible for care plans and reviews.		x		The information resource aims to provide general guidance applicable across diverse care settings. Specifying a particular name or professional role within this resource would be misleading, as these details are context‐specific to each care home's operational framework and the multi‐disciplinary team involved. The revised text includes that assessments should be conducted by individuals with the required levels of skills and knowledge for the particular task, acknowledging the varied professional roles. The scope of this document primarily focuses on the principles of care planning rather than the granular details of operational responsibility for care provision within specific settings. Further detail on the individual professionals responsible would typically be found within the resident's specific care plan and the care home's internal policies, which are beyond the remit of this general information resource.
Refer to instances when a lasting power of attorney is not in place.		x		If a lasting power of attorney is not in place, a family/friend may be eligible to apply for a deputy appointed by the Court of Protections. Information about becoming someone's deputy and the link to the government website has been provided.
Include information about medication, dietary needs, allergies and intolerance, risk management key people, attorneys, GP and local authority in the contents of a care plan.	x			Included within the section entitled ‘What Goes into a Care Plan?’
Include a sentence stating that professionals may include care home staff, family members and external medical professionals.	x			This has been included.
Include that the care plan may be shared with relevant health and social care professionals outside of the care home.	x			This has been included.
Include how to check that the care plan is being implemented			x	Beyond the scope of the study.
Remind family/friends that supporting a person move into a care home is often an emotionally challenging period, and involvement in care planning will likely take time.	x			This has been included.
Link to what a ‘good’ care plan looks like			x	Care plans are individual and should be specific to each person.
Link to further information which may help family/friends to support an older adult move into a care home		x		Links to information and advice provided by a well‐known charity (Age UK) in the England has been included.
Include how to raise and report complaints		x		A link to a well‐known charity in England (Age UK) has been included. The link outlines steps to undertake to raise/report complaints relating to funded and/or self‐funded care in care homes.
Discrepancy in terminology—‘a person’ and ‘the person’.	x			Amended accordingly.
Lacking humanity		x		The document's language and tone were developed and reviewed by two public advisors with lived experience of supporting family members in older adult care settings. Their direct involvement aimed to ensure the content is not only informative but also sensitive and empathetic to the target audience. While defining ‘humanity’ in a document can be subjective, the current iteration reflects a concerted effort to balance clarity and compassion for broad public dissemination. We will continue to evaluate opportunities for further refinement in subsequent versions.
Change phrase ‘If it is anticipated that someone's condition will deteriorate in the future…’ to ‘in anticipation of someone's condition deteriorating in the future…’	x			Amended accordingly.
Add examples of questions to ask oneself to guide family/friends as to the types of information to include in a care plan/provide to care home staff		x		This would increase the length of the information resource. The section ‘What goes into a Care Plan’ has been revised which may help to identify the types of information to provide to staff.
Include more information/some information included does not need explaining			x	The information resource has sought to be applicable to people who are experiencing a wide range of experiences and so has aimed strike a balance between concision and depth.

Suggested improvements included adding information on the legal requirements of care homes concerning care planning, including resources to help support a person move into a care home and signposting readers on how to raise complaints.

I think it would be really, really helpful to have some information at the bottom that says, these are the things that should be happening, if they're not happening, or they're not happening to a satisfactory level, what do you do, who do you go to?

FG2, P3

However, participants held mixed views regarding the length and layout of the information resource. Some participants found the document to be of a suitable length, others found it ‘a bit light’ (FG3, P5) or ‘…too long and (un)wieldy’ (FG4, P4). Some participants found that the information resource was not engaging enough, and would benefit from including diagrams, pictures, bolder colours and/or categorising the content.

…I think it would be great if it was a bit more visual, with some quite kind of clear call to actions in it, and kind of categorised, whether that's kind of colour code categorisation, or blocks of images with text overlaid.

FG2, P2

Others commented that efforts to make the document more engaging may lengthen it and risked making it less accessible.

…it's always tempting to prettify these documents, but I think in this case the fact that it is just two sides of A4 [paper] is a definite bonus.

FG1, P5

### 3.5 Sharing the Information Resource

When asked, several participants reported that, subject to some changes (Table 2), they would share the information resource amongst family/friends of an older adult residing in a care home.

But listening to people, and local people that I've spoken to, there's a lot of things that, you know, they're not made aware of in other [care] homes. So, I definitely would share.

FG2, P6

However, some participants would not share the information resource. The current presentation of the information resource formed the most reported reason, and one participant commented that they would not share the document as it ‘… doesn't seem human enough…’ (FG2, P4)

…I wouldn't share this particular document…this really, really needs to have impact, so it needs to be something bold, interesting, friendly, succinct.

FG2, P3

### 3.6 Access to the Information Resource

The importance of facilitating access to the information resource was discussed. Suggestions to promote access included engaging with relevant charities, the availability of digital and nondigital formats, and incorporating it in online directories of local care homes.

I also for a while was [working in a charity] … signposting to things like this [information resource] would be really helpful.

FG2, P2

Some participants felt that the information resource could be included within an App, training package, toolkit or platform, that encompasses relevant information, including care planning, to help family/friends to support an older adult move into a care home.

…I think a sort of package would be really good, a sort of toolkit like P2 said, and something that would be really good to help people with those transitions…I think it's a difficult journey, and I think the care plan is just one part of that journey…

FG2, P4

Moreover, it was suggested that the information resource could be made available to health and social care professionals, including paramedics, to share with family/friends of an older adult where a possible need to move into a care home has been identified.

So, this is before a care home is even thought about, it might be ambulance staff picking up… that they can see that possibly there will be a need for this person to go in a care home… They [paramedics] can say, ‘…there's this really good document [information resource] …’

FG4, P2

Several participants described the stressful and emotional period of supporting the move of a family member/friend into a care home. While participants would have valued receiving a document akin to the information resource, they commented that, they may not have been receptive to it at that point and suggested that family/friends would need to be encouraged to return to it in the future.

But I think at the point of taking someone in, it [information resource] will almost go in one ear and straight out the other… So, it's great to have it [information resource] and be able to refer to it [information resource], but it also needs to be revisited, because I just don't think you're in an emotional state to cope with yet another bit of paper…

FG4, P7

## 4 Discussion

This is the first study to explore and incorporate family/friends' views on the relevance and usefulness of a draft information resource focusing on care planning to address and support the needs of older adults living in a care home in England.

We found heterogeneity in participants' experiences in care planning. Some participants shared experiences that embodied a person‐centred approach, however, others commented on a lack of involvement. This is consistent with a recent study that suggests that the level of family/friend involvement in care planning varies across care homes, with some health and social care professionals reporting that family/friend contribution to care plans was uncommon [12]. Challenges to involving family/friends in care planning have been reported and include limited staff training [12, 13]. The development of ‘key principles’ to care planning in care homes for health and social professionals [15, 16], which include the involvement of family/friends, provides an opportunity to inform and possibly improve practice.

Our analysis suggests that most family/friends of an older adult residing in a care home would value an information resource on care planning and that such a document could help to improve their involvement in the process. Acknowledging that previous research has largely focused on interventions to promote involvement in ACP, as opposed to care planning to address the current needs of older adults living in care homes [23], we draw on ACP literature to discuss our participants' views on our information resource. Prior to supporting an older adult to move into a care home, most participants in our study were unfamiliar with the concept of care planning, which broadly aligns with a scoping review which found the public knowledge on ACP is generally low [40]. The information resource we devised was deemed useful for introducing and highlighting the importance of care planning. This resonates with Sussman and colleagues' study [28], which suggests that an information resource could increase family/friends' understanding of ACP. This is important as limited awareness has been identified as a barrier to participating in ACP conversations [22]. In addition to increasing awareness, participants in our study felt that the information resource instilled a sense of empowerment that could foster greater family/friend involvement in care planning. Cognisant of the importance of involving family/friends in care planning to deliver person‐centred care and support [12, 13], a suitable information resource could promote awareness of and involvement in care planning. Nevertheless, further research is required to evaluate whether the information resource improves actual or perceived involvement of family/friends in care planning and to ascertain its potential contribution to the delivery of person‐centred care.

Overall, participants in our study were satisfied with the contents of the information resource and found the document easy to understand. People had different opinions, however, regarding the document's presentation, amount of information, use of colour and images. Van de Steen and colleagues [27] explored the views of family members of a person living in a care home on an information resource on comfort care in three countries. Variation in preferences of how much information to include was found among family members [27], which broadly aligns with our findings. Moreover, contrasting views relating to the design of health‐related information resources have also been reported in some studies [38, 41]. However, in these studies, service users and healthcare professionals fed back on draft information resources that had been visually designed [38, 41], whereas our document was presented as a Word document, without illustrations. Revising the presentation of our information resource forms part of future planned work, and it will be important to address and incorporate our participants' differing views to promote the acceptability and usability of the document. Furthermore, it is important to consider strategies that facilitate access to and use of information resources [42]. Much like in Dupont and colleagues' study [43] that developed an ACP website, participants in our study highlighted the importance of disseminating the information resource by digital and nondigital media and liaising with established third sector organisations to promote uptake.

## 5 Limitations

Owing to limited resources, focus groups were held remotely, which may have discouraged the participation of people who experience digital exclusion [44]. However, remote approaches can offer convenience, which may have facilitated the participation of family/friends with other responsibilities. Secondly, while some participants did interact with one another, most interaction occurred with the facilitator(s). Reducing the number of participants per group discussion may have fostered greater interaction amongst participants [45] but may also have narrowed the breadth and diversity of opinions expressed in the group. Third, we did not collect information on participants' educational backgrounds. Previous research has demonstrated how perceptions of care‐related information resources can vary by educational attainment [28], and further evaluation of our information resource may benefit from exploring the acceptability of the document among individuals with diverse educational backgrounds. Fourth, we did not collect data on whether participants had experience of care planning beyond supporting an older adult residing in a care home in England. It is important to acknowledge that participants' professional and/or personal experiences (including having lived in a care home or supporting a family member/friend in care homes outside England) may have influenced their views on our information resource. Therefore, any subsequent evaluations would benefit from collecting feedback from individuals with a diverse range of professional and personal experiences to understand the resource's impact on those with varying levels of prior exposure. Fifth, the draft information resource was informed by previous research involving social care professionals working within the care home sector [12, 15, 16, 23]. Two public advisors with experience of supporting an older adult in a care home advised on the development and revision of the information resource. However, a limitation of the study is the absence of iterative member‐checking, which is a key tool for enhancing the trustworthiness and validity of qualitative findings [46]. While feedback was used to refine the information resource, a more recursive, dialogic approach might have allowed for a deeper co‐construction of meaning, thereby further strengthening the credibility of the thematic interpretations [47]. Sixth, a convenience approach to sampling was employed due to limited time and resources. This has implications to the representativeness of our sample, with subsequent limitations to the transferability of our findings. While we did not collect information on participants' family member/friend's health condition(s), most of our participants were recruited through Join Dementia Research. We sought to develop an information resource on care planning in general, however, acknowledge that some health conditions may influence the level of family/friend involvement in care planning, and reporting on participants' family member/friend's health condition(s) would have benefitted the transferability of our findings. Moreover, our sample were largely from a White ethnic background, and it is important that future research explores the views of family/friends from diverse ethnic groups. Lastly, questions guiding the focus group discussions focused on participants' views of the information resource. Nevertheless, participants shared their experiences, which have been reported here. However, future research would benefit from gaining a greater understanding of family/friends' experiences in care planning in older adult care homes.

## 6 Conclusions

This is the first study to explore and incorporate on family/friends' views on a draft information resource seeking to promote their involvement in care planning focusing on addressing the needs of older adults residing in care homes in England. Most participants believed that the resource was helpful and felt that it had the potential to facilitate family/friend engagement in the care planning. Further exploration is required to understand the potential impact of the information resource on family/friend involvement in the care planning process.

## Author Contributions

**Thaïs Caprioli:** conceptualisation, investigation, writing – original draft, writing – review and editing, data curation, formal analysis. **Jonathan Taylor:** conceptualisation, investigation, writing – review and editing, data curation, formal analysis. **Yuri Hamashima:** conceptualisation, investigation, writing – review and editing, formal analysis, data curation. **Alison Charles:** conceptualisation, investigation, writing – review and editing, formal analysis, data curation. **Jacqueline Damant:** conceptualisation, writing – review and editing. **Clarissa Giebel:** conceptualisation, writing – review and editing. **Michele Peters:** conceptualisation, writing – review and editing. **Madalina Toma:** conceptualisation, writing – review and editing. **Anna Ferguson Montague:** conceptualisation, writing – review and editing. **Lynne Wright:** conceptualisation, writing – review and editing. **Nick Smith:** conceptualisation, funding acquisition, formal analysis, project administration, writing – review and editing.

## Conflicts of Interest

The authors declare no conflicts of interest.

## Supporting information

Supporting File

## Data Availability

The data that support the findings of this study are openly available in the UK Data Service ReShare at https://reshare.ukdataservice.ac.uk/858042/. (https://ukdataservice.ac.uk/).
