# Supplementary material for: Promoting Family/Friend Involvement in Care Planning in Care Homes: A Qualitative Exploration of the Usefulness and Relevance of an Information Resource
Source: Health Expect. 2026 Jun 4;29(3):e70715. doi: 10.1111/hex.70715 (PMC13250389; doi:10.1111/hex.70715)
Supplement: Supplementary file 1 — Supporting File [file HEX-29-e70715-s001.docx]

#### **Supplementary File 1: Completed Standard for Reporting Qualitative Research (SRQR) Checklist (O’Brien et al., 2014)**

| **Item** | **Section** | **Additional information** |
| --- | --- | --- |
| **Title and abstract** | | |
| **Title** - Concise description of the nature and topic of the study Identifying the study as qualitative or indicating the approach (e.g., ethnography, grounded theory) or data collection methods (e.g., interview, focus group) is recommended | Title (p.1) |  |
| **Abstract** - Summary of key elements of the study using the abstract format of the intended publication; typically includes background, purpose, methods, results, and conclusions | Abstract (p.1) |  |
| **Introduction** | | |
| **Problem formulation** - Description and significance of the problem/phenomenon studied; review of relevant theory and empirical work; problem statement | Background (p.2-3) |  |
| **Purpose or research question** - Purpose of the study and specific objectives or questions | Background (p.3) |  |
| **Methods** | | |
| **Qualitative approach and research paradigm** – Qualitative approach (e.g., ethnography, grounded theory, case study, phenomenology, narrative research) and guiding theory if appropriate; identifying the research paradigm (e.g., postpositivist, constructivist/ interpretivist) is also recommended; rationale* | Methods (p.3) |  |
| **Researcher characteristics and reflexivity** - Researchers’ characteristics that may influence the research, including personal attributes, qualifications/experience, relationship with participants, assumptions, and/or presuppositions; potential or actual interaction between researchers’ characteristics and the research questions, approach, methods, results, and/or transferability | See additional information | The four researchers involved in data collection and analysis were academic researchers. They were early career researchers, with relevant postgraduate qualification and experience in social care research. Guidance from experienced researchers was provided throughout. The remaining authors who were involved and advised on the study were senior researchers or PPIE advisors. |
| **Context** - Setting/site and salient contextual factors; rationale* | Participants and recruitment (p.4) |  |
| **Sampling strategy** - How and why research participants, documents, or events were selected; criteria for deciding when no further sampling was necessary (e.g., sampling saturation);  rationale* | Participants and recruitment (p.4) |  |
| **Ethical issues pertaining to human subjects** -  Documentation of approval by an appropriate ethics review board and participant consent, or  explanation for lack thereof; other confidentiality and data security issues | Ethics (p.6) |  |
| **Data collection methods** - Types of data collected; details of data collection procedures including (as appropriate) start and stop dates of data collection and analysis, iterative process, triangulation of sources/methods, and modification of procedures in response to evolving study findings; rationale* | Data collection (p.5) |  |
| **Data collection instruments and technologies** – Description of instruments (e.g., interview guides, questionnaires) and devices (e.g., audio recorders) used for data collection; if/how the instrument(s) changed over the course of the study | Data collection (p.5) |  |
| **Units of study** - Number and relevant characteristics of participants, documents, or events included in the study; level of participation (could be reported in results**)** | Findings (p.6-7) |  |
| **Data processing** - Methods for processing data prior to and during analysis, including transcription, data entry, data management and security, verification of data integrity, data coding, and anonymization/de-identification of excerpts | Data collection; Data analysis  (p.5-6) |  |
| **Data analysis -** Process by which inferences, themes, etc., were identified and developed, including the researchers involved in data analysis; usually references a specific paradigm or approach; rationale* | Data analysis  (p.5-6) |  |
| **Techniques to enhance trustworthiness -** Techniques to enhance trustworthiness and credibility of data analysis (e.g., member checking, audit trail, triangulation); rationale* | Data analysis  (p.5-6) |  |
| **Results / findings** | | |
| **Synthesis and interpretation -** Main findings (e.g., interpretations, inferences, and themes); might include development of a theory or model, or integration with prior research or theory | Findings  (p.6-12) |  |
| **Links to empirical data -** Evidence (e.g., quotes, field notes, text excerpts, photographs) to substantiate analytic findings | Findings  (p.6-13) |  |
| **Discussion** | | |
| **Integration with prior work, implications, transferability, and contribution(s) to the field -** Short summary of main findings; explanation of how findings and conclusions connect to, support, elaborate on, or challenge conclusions of earlier scholarship; discussion of scope of application/generalizability; identification of unique contribution(s) to scholarship in a discipline or field | Discussion  (p.13-14) |  |
| **Limitations -** Trustworthiness and limitations of findings | Limitations  (p.14-15) |  |
| **Other** | | |
| **Conflicts of interest -** Potential sources of influence or perceived influence on study conduct and conclusions; how these were managed | End of manuscript  (p.15) |  |
| **Funding -** Sources of funding and other support; role of funders in data collection, interpretation, and reporting | End of manuscript  (p.15) |  |

* The rationale should briefly discuss the justification for choosing that theory, approach, method, or technique rather than other options available, the assumptions and limitations implicit in those choices, and how those choices influence study conclusions and transferability. As appropriate, the rationale for several items might be discussed together.

#### **Supplementary File 2: Draft information resource**

Understanding Care Planning in Care Homes for Older Adults in England and How You Can Contribute: A Guide for Family and Friends

This guide is designed to help you understand care planning in care homes for older adults in England and how you can contribute to the process.

**What is a Care Plan?**

A care plan is a document that helps to ensure a person receives high-quality care that respects their wishes and meets their needs. It's an ever-changing document that helps everyone involved in a person’s care understand who they are, what matters to them, and how to support them to live a fulfilling life.

**Why is it Important?**

- Person-centred care: It helps tailor care to a person’s unique preferences, abilities, and needs.
- Transparency and continuity: It ensures that all health and care professionals involved know how to provide the best support for each person’s needs.
- Empowerment: It gives a person as much choice and control as is possible over their daily life.

**What Goes into a Care Plan?**

In addition to information about day-to-day care preferences and needs, a care plan typically includes information about a person’s:

- History, background, friends and family
- Capabilities
- Preferred ways of being supported
- Hobbies, interests, and aspirations
- End-of-life care preferences.

**Why your Input Matters**

As a family member or friend, you may wish to contribute to the care planning process by:

- Sharing insights: You may know the person’s life history, interests, and values. This information can help the care team understand what matters most to them and how best to support them.
- Supporting communication: If the person has difficulty expressing themselves, you can help communicate their preferences.
- Participating in reviews: Care plans are reviewed regularly. With the person’s consent, you may be invited to these reviews to provide input.
- Helping to ensure that a person’s end of life preferences, including funeral arrangements (which may be documented in an advance care plan), are respected. This may involve helping the care home to complete a DNACPR or ReSPECT form.

Your level of involvement may vary over time based on changes in a person’s health or their capacity to advocate for themselves

**When are Care Plans Developed and Updated?**

Care plans are ever-changing documents and will be updated regularly. You may wish to contribute to ensure that the information is accurate. Care plans are often developed:

- Prior to arrival: Information collected at this stage, which may be provided by a social worker, hospital, GP and/or former care home, is often used as the foundation of a person’s care plan.
- On arrival at the care home: A care plan is developed shortly after admission, when staff will gather as much information as possible from the person, their clinicians, and, with consent, from family and friends.
- During regular reviews: Reviews often occur every 4-6 weeks or in response to significant changes in health, wellbeing, or preferences.

**Viewing a care plan**

The person has the right to see their care plan. If you are appointed an attorney, under the terms of a Lasting Power of Attorney (LPA), you can also request to view the sections of the person’s care plan for which you have power of attorney, e.g. health and welfare, or financial matters.

If you have not been appointed an Attorney, the person can consent for you to be given access to certain sections of the care plan.

**Glossary**

Advance Care Plan (ACP): An ACP relates to future care provision and is often focused on end-of-life care. ACPs are often developed if it is anticipated that someone’s condition will deteriorate in the future.

DNACPR: Do Not Attempt Cardio Pulmonary Resuscitation (CPR) - a form that records a person's wish not to receive CPR if their heart stops beating or they stop breathing.

ReSPECT Form: Recommended Summary Plan for Emergency Care and Treatment - a form that records what types of care and treatment a person would or would not want in an emergency.

Lasting Power of Attorney (LPA): This is a legal document that lets a person appoint one or more people (known as ‘attorneys’) to help them make decisions or to make decisions on their behalf. There are two types of LPAs:

1. Health and welfare.
2. Property and financial affairs.

A person may have one or both types of LPA in place.

Person-Centred Care: An approach that puts the person at the centre of their care planning, focusing on their unique needs, preferences, and wishes, rather than standard routines.

Wellbeing: A broad concept that includes physical health, mental health, emotional state, social relationships, and overall quality of life.

#### **Supplementary File 3: Topic guide**

1. Is there anything you wished you had been told about care plans before your friend/family member went into a care home?
2. Does this document help to answer these questions?
3. Do you think a document of some kind could help family and friends to understand care planning?
   - If yes, why would this be helpful? / If yes, what should be included in this document? / If no, what would be helpful?
4. Is this document easy to understand?
   - If no, why not? / If no, how could it be made easier to understand?
5. What do you find helpful in this document?
6. Could this document be improved?
   - If yes, how? [content, topics, layout structure, use of images]
7. If someone you know has a friend or family member who has recently begun living in a care home, would you share a document of this kind with them?
   - If yes, why? / If no, why not? / If no, what might encourage you to do so?

Possible prompts

- - Are there particular words or phrases that you feel should be changed? [“The person” vs. “Your loved one”
  - Would it be helpful to include some information about digital care plans?
  - When we talk about “everyone involved in a person’s care”, would it be helpful to be provided with some examples?

1. Do you have any final comments?

#### **Supplementary File 4: Table outlining the relationship between participants and the person (s) they have / are supporting**

|  | **Participant number** | **Supporting / supported their** |
| --- | --- | --- |
| **FG1** | | |
|  | 1 | Grandfather |
|  | 2 | Father |
|  | 3 | Mother |
|  | 4 | Father |
|  | 5 | Father |
|  | 6 | Husband’s Aunt |
|  | 7 | Grandmother |
| **FG2** | | |
|  | 1 | Uncle |
|  | 2 | Father |
|  | 3 | Mother |
|  | 4 | Mother |
|  | 5 | Mother |
|  | 6 | Husband’s Aunt |
|  | 7 | Wife |
| **FG3** | | |
|  | 1 | Two aunts |
|  | 2 | Mother |
|  | 3 | Sister |
|  | 4 | Mother |
|  | 5 | Father, Friend |
|  | 6 | Uncle, Friend’s Father |
|  | 7 | Mother |
| **FG4** | | |
|  | 1 | Mother |
|  | 2 | Mother |
|  | 3 | Mother |
|  | 4 | Father |
|  | 5 | Father |
|  | 6 | Mother |
|  | 7 | Husband |

FG: focus group
